# The Role of *TLR4* 896 A>*G* and 1196 C>*T* in Susceptibility to Infections: A Review and Meta-Analysis of Genetic Association Studies

**DOI:** 10.1371/journal.pone.0081047

**Published:** 2013-11-25

**Authors:** Panayiotis D. Ziakas, Michael L. Prodromou, Joseph El Khoury, Elias Zintzaras, Eleftherios Mylonakis

**Affiliations:** 1 Division of Infectious Diseases, Rhode Island Hospital, Providence, Rhode Island, United States of America; 2 Warren Alpert Medical School of Brown University, Providence, Rhode Island, United States of America; 3 Department of Medicine, Warren Alpert Medical School of Brown University, Providence, Rhode Island, United States of America; 4 Center for Immunology and Inflammatory Diseases and Division of Rheumatology, Allergy, and Immunology, Massachusetts General Hospital and Harvard Medical School, Charlestown, Massachusetts,United States of America; 5 Division of Infectious Diseases, Massachusetts General Hospital, Boston, Massachusetts, United States of America; 6 Center for Clinical Evidence Synthesis, Institute for Clinical Research and Health Policy Studies, Tufts Medical Center, Boston, Massachusetts, United States of America; 7 Department of Biomathematics, School of Medicine, University of Thessaly, Larissa, Greece; University of Milan, Italy

## Abstract

**Background:**

Toll-like receptor 4 plays a role in pathogen recognition, and common polymorphisms may alter host susceptibility to infectious diseases.

**Purpose:**

To review the association of two common polymorphisms (*TLR4* 896*A*>*G* and *TLR4* 1196C>*T*) with infectious diseases.

**Data Sources:**

We searched PubMed and EMBASE up to March 2013 for pertinent literature in English, and complemented search with references lists of eligible studies.

**Study Selection:**

We included all studies that: reported an infectious outcome; had a case-control design and reported the *TLR4* 896*A*>*G* and/or *TLR4* 1196C>*T* genotype frequencies; 59 studies fulfilled these criteria and were analyzed.

**Data Extraction:**

Two authors independently extracted study data.

**Data Synthesis:**

The generalized odds ratio metric (OR_G_) was used to quantify the impact of *TLR4* variants on disease susceptibility. A meta-analysis was undertaken for outcomes reported in >1 study. Eleven of 37 distinct outcomes were significant. *TLR4* 896 A>*G* increased risk for all parasitic infections (OR_G_ 1.59; 95%CI 1.05-2.42), malaria (1.31; 95%CI 1.04-1.66), brucellosis (2.66; 95%CI 1.66-4.27), cutaneous leishmaniasis (7.22; 95%CI 1.91-27.29), neurocysticercosis (4.39; 95%CI 2.53-7.61), *Streptococcus pyogenes* tonsillar disease (2.93; 95%CI 1.24-6.93) , typhoid fever (2.51; 95%CI 1.18-5.34) and adult urinary tract infections (1.98; 95%CI 1.04-3.98), but was protective for leprosy (0.36; 95%CI 0.22-0.60). *TLR4* 1196 C>*T* effects were similar to *TLR4* 896 A>*G* for brucellosis, cutaneous leishmaniasis, leprosy, typhoid fever and *S. pyogenes* tonsillar disease, and was protective for bacterial vaginosis in pregnancy (0.55; 95%CI 0.31-0.98) and *Haemophilus influenzae* tonsillar disease (0.42; 95%CI 0.17-1.00). The majority of significant associations were among predominantly Asian populations and significant associations were rare among European populations.

**Conclusions:**

Depending on the type of infection and population, TLR4 polymorphisms are associated with increased, decreased or no difference in infectious disease. This may be due to differential functional expression of TLR4, the co-segregation of TLR4 variants or a favorable inflammatory response.

## Introduction

Toll-like receptors (TLRs) are a class of highly conserved membrane bound pattern recognition receptors (PRRs) that play an integral role in the regulation of the immune system through the recognition of pathogen-associated molecular patterns (PAMPs) and the activation of immune response genes [1,2]. Toll-like receptor 4 (TLR4), is a well-studied TLR, specifically recognizing lipopolysaccharide from Gram-negative bacteria [3,4] and initiating intracellular signal cascades, that involve the adaptor protein encoded by the myeloid differentiation primary response gene 88 (MyD88), which ultimately activates nuclear factor kappa B [5] and leads to interferon production [6]. TLR4 has also been shown to recognize mannans of fungal pathogens [7], *Mycobacterium tuberculosis* [8], and the fusion protein of respiratory syncytial virus [9]. 

 Two single nucleotide polymorphisms (SNPs), *TLR4* 896 A>*G* (corresponding to an Asp299Gly substitution mutation ; SNP ID: rs 4986790) and *TLR4* 1196 C>*T* (corresponding to a Thr399Ile substitution mutation; SNP ID: rs 4986791), have been shown to be associated with LPS hyporesponsiveness [10,11]. In whites, the two SNPs are in linkage disequilibrium (D=1 and r2=0.791, HapMap accessible at: http://hapmap.ncbi.nlm.nih.gov/). Structurally, these mutations are found outside of the ligand binding domain of TLR4 and crystal structures have shown that these mutations have no effect on LPS binding. Instead, they do cause local conformational changes around the area of the mutation that may affect folding efficiency, cell surface expression, protein stability, as well as interaction with downstream messenger proteins [12]. At the molecular level, it has been shown that the *TLR4* 896 A>*G* mutation interferes with TLR4 interaction with MyD88 and other downstream messengers [13]. These mutations also appear to affect the levels of functional TLR4 expression, leading to a 2-fold reduction [14]. This reduction is further amplified to 10-fold in the absence of myeloid differentiation factor 2 (MD-2) which forms a complex with TLR4 and LPS [14,15].

There has been great interest regarding the association of the TLR4 SNPs *TLR4* 896 A>*G* and *TLR4* 1196 C>*T* to susceptibility for infection and other non-infectious disease states. Clinical studies associating these SNPs to infectious disease susceptibility have produced mixed results [16-19]. The present study aims to reassess the association of *TLR4* 896 A>*G* and *TLR4* 1196 C>*T* with infectious disease susceptibility using the Generalized Odds Ratio (OR_G_), which can elucidate the magnitude and association of individual genotypes with susceptibility to disease [20].

## Materials and Methods

### Study Selection

We conducted searches on Pubmed and EMBASE up to March, 2013 (last access on March 3, 2013). The search terms included: “(toll AND like AND receptor AND 4 AND polymorphism) OR (TLR4 AND polymorphism) OR Asp299Gly OR D299G OR Thr399Ile OR T399I” for PubMED; “('tlr4'/exp OR 'tlr4') AND ('receptor'/exp OR 'receptor') AND (polymorphism OR asp299gly OR d299g OR thr399ile OR t399i)” for EMBASE. The titles and abstracts of the studies were reviewed; titles that included TLR4 polymorphisms and risk for infectious disease were included for more detailed evaluation. Studies that reviewed TLR4 polymorphisms and their association with non-infectious disease were excluded, as were studies that were not published in English. An eligible study fulfilled all of the following three criteria: (i) the study reported an infectious disease outcome, (ii) the study was performed using a case-control design, where “cases” refer to subjects with a disease outcome and controls refer to a healthy population (without the disease outcome), and, (iii) the study reported genotype frequencies for *TLR4* 896 A>*C*, *TLR4* 1196C>*T*, or both.

### Data Extraction

Two authors (PDZ and MLP) independently extracted data from the final included articles. Any discrepancies were reviewed and resolved by consensus. The information extracted included name of first author, origin of population being studied, number of cases and controls being studied subdivided by genotype frequencies (homozygous wild-type, heterozygous, and homozygous mutant), the disease being studied, and the conclusions reportedly drawn from each study.

### Data Synthesis

We used the generalized odds ratio (OR_G_) along with its 95% Confidence Interval (95% CI) to address the association of *TLR4* 896 A>*C* and *TLR4* 1196 C>*T* polymorphisms with outcomes of interest (disease susceptibility). The OR_G_ provides a model-free approach of estimating the genetic risk in genetic association studies (GAS) and meta-analysis of GAS, depending on the mutational load [20]. The OR_G_ is defined as follows: for any two subjects, one diseased (case) and one non-diseased (control), the OR_G_ estimates the odds of being diseased relative to the odds of being non-diseased when the diseased subject has higher mutational load than the non-diseased subject, i.e. the risk of disease is proportional to the increased genetic exposure. Alternatively, the OR_G_ shows how many diseased-healthy pairs exist in the study for which the diseased have the larger mutational load, relative to the number of pairs for which the non-diseased have the larger mutational load [20][21]. The OR_G_ estimates the overall genetic risk effect by utilizing the complete genotype distribution whereas the OR of conventional genetic models (additive, dominant, recessive, co-dominant) is calculated by merging genotypes. In addition, the conventional genetic models are not independent and thus, the interpretation of results is difficult when more than one model is significant [22]. In the meta-analysis of GAS, heterogeneity was quantified using the Cochran’s Q and I^2^ metric [20]. The existence of the differential magnitude of effect in large versus small studies was checked using the Harbord’s test [23] for meta-analysis involving at least four studies. Also, the Hardy-Weinberg equilibrium (HWE) was used as a quality criterion for control populations. HWE deviations may result in biased estimations as they can influence type-I error in single study effects, and may alter statistical significance in meta-analysis of gene-disease associations [24,25]. The HWE deviations amongst the control populations were screened using the chi-square test [26]. For single studies deviating from HWE, a sensitivity analysis was performed after correction of control group with the expected genotype frequencies [22,27]. ORG was calculated using the ORGGASMA application available at http://biomath.med.uth.gr [20]. This study complies with the PRISMA guidelines for reporting reviews and meta-analyses (Checklist S1) [28].

## Results

A total of 962 studies from PubMed and 1615 from EMBASE were initially retrieved, comprising a total of 2,197 non-duplicate studies (Table S1-Flow diagram). After reading the title and the abstract, 117 studies were found to be suitable for further evaluation. Of the 117 articles reviewed in detail, 58 studies were excluded (18 studies did not publish genotypic frequencies, 13 had no healthy controls in their experimental design, 5 focused on in vitro functional studies, 8 did not study the desired polymorphisms, 6 were either reviews or a meta-analysis, and 8 studies had non extractable data for other reasons). A total of 59 case-control studies [29-87] were included in the analysis, reporting 37 different disease outcomes (Tables 1 and 2). The origin of studies was in descending order Europe (28 studies), Asia (12 studies), South America (7 studies), Africa (6 studies), North America (5 studies), Australia (1 studies). 

**Table 1:** Genotypic frequencies reported for the *TLR4* 896 A>*G* SNP and association with disease outcome; significant effects are in bold; outcomes that have been studied more than once have been grouped together in the table, with the overall effect described in the shaded area^***†***^ genotypic frequencies of controls that did not satisfy Hardy Weinberg Equilibrium, [effects in brackets after correction of HWE deviations].

		Control Genotype			Case Genotype					
**Name**	**Population**	**A/A**	**A/G**	**G/G**	**A/A**	**A/G**	**G/G**	**Disease Outcome**	**Conclusion Reported**	**OR_G_ (95% CI)**
Carvalho et al [29]	England	70	10	0	58	18	0	Aspergillosis	Overall susceptibility not studied	2.10 (0.92-4.81)
Rezazadeh et al [30]	Iran	65	46	0	68	127	3	Brucellosis	Increased risk**^*†*^**	2.66(1.66-4.27) [**2.69 (1.67-4.33**)]
Doorduyn et al [31]	Netherlands	608	72	3	405	49	1	Campylobacter	No association	1.00 (0.68-1.46)
Plantinga et al [32]	Tanzania	99	9	0	107	10	0	Oropharyngeal candidiasis in HIV	No association	1.02(0.41-2.55)
Laisk et al [33]	Estonia	287	35	1	61	9	0	*C.trachomatis*(women)	No association	1.24 (0.58-2.67)
Szebeni et al [34]	Hungary	108	10	0	37	4	0	NecEnterocolitis in LBW infants	No association	1.26 (0.40-4.00)
Lee, et al [35]	United States	431	11	21	103	2	3	Gram –ve infections in liver transplant	No association**^*†*^**	0.66 (0.26-1.70) [0.42(0.16-1.66)]
Ajdary, et al [36]	Iran	73	2	0	102	26	0	Leishmaniasis (Cutaneous)	Increased risk	**7.22 (1.91-27.29)**
Rasouli et al [37]	Iran	137	18	0	110	11	1	Leishmaniasis (Visceral)	No asscociation	0.81 (0.38-1.75)
Bochud et al [38]	East Africa	155	37	2	375	32	2	Leprosy	Protective	0.36 (0.22-0.60)
West, et al [39]	Thailand	1377	20	1	484	5	0	Meliodosis	No association**^*†*^**	0.74(0.29-1.92) [0.70(0.27-1.78)]
Verma, et al [40]	India	127	22	1	77	61	2	Neurocysticercosis	Increased risk	**4.39(2.53-7.61)**
Montes et al [41]	Spain	135	20	0	65	12	3	Osteomyelitis	Increased risk	1.55 (0.76-3.20)
Emonts et al [42]	Netherlands	374	58	1	293	42	2	Otitis media (acute)	Overall susceptibility not studied	0.96 (0.63-1.45)
Moens et al [43]	Belgium	161	16	1	84	13	2	Invasive pneumococcal infection	No association	1.69 (0.81-3.54)
Mrazek et al [44]	Czechoslovakia	217	34	1	89	9	0	Prosthetic joint infection	No association	0.66 (0.31-1.42)
Doorduyn et al [31]	Netherlands	608	72	3	173	20	0	*Salmonella* gastroenteritis	No association	0.96 (0.57-1.60)
Yuan et al [45]	Australia	364	44	1	82	3	0	*S. pneumoniae*	Protective	0.35 (0.12-1.07)
Liadaki, et al [46]	Greece	195	27	0	99	6	0	Tonsillar Disease (*H.influenzae*)	No association	0.47 (0.19-1.14)
Liadaki, et al [46]	Greece	264	25	0	30	8	0	Tonsillar Disease (*S.pyogenes*)	Increased risk	**2.93 (1.24-6.93)**
Bhuvanendran, et al [47]	Malaysia	241	9	0	277	27	0	Typhoid Fever	Increased Risk	**2.51 (1.18-5.34)**
Yin, et al [48]	China	227	21	0	109	20	0	UTI (Adults)	Increased risk	**1.98 (1.04-3.98)**
Hawn et al [49]	United States	274	33	6	585	65	2	UTI (Women)	No association	0.79 (0.52-1.20)
								**Chagas Disease**		1.06 (0.53-2.14)
Weitzel, et al [50]	Northern Chile	42	3	0	114	11	0	Chagas Disease	No association	1.20 (0.35-4.14)
Zafra et al [51]	Colombia	191	9	0	262	10	3	Chagas Disease	No association	1.00 (0.43-2.36)
								***H. pylori***		0.91 (0.61-1.36)
Achyut et al [52]	India	168	32	0	110	20	0	*H. pylori*	No association	0.97 (0.53-1.76)
Moura et al [53]	Brazil	222	28	4	206	25	1	*H. pylori*	No association**^*†*^**	0.87(0.50-1.50) [0.81(0.47-1.40)]
								**Malaria**		**1.31 (1.04-1.66)**
Esposito, et al [54]	Burundi	300	36	1	528	72	2	Malaria (children)	No association	1.13 (0.74-1.73)
Zakeri, et al [55]	Iran	287	33	0	276	39	5	Malaria (all ages)	No association	1.38 (0.86-2.22)
Mockenhaupt et al [56]	Ghana	239	47	4	444	129	7	Malaria (pregnancy)	Overall susceptibility not studied	1.42 (0.99-2.02)
								**Meningococcal disease**		1.10 (0.90-1.34)
Biebl et al [57]	Austria	678	88	3	167	18	0	Meningococcal disease (all ages)	No association	0.82 (0.49-1.40)
Read et al [58]	England	787	81	11	924	110	13	Meningococcal disease (all ages)	No association**^*†*^**	1.13 (0.86-1.51) [1.05(0.79-1.38)]
Faber et al [59]	Europe	190	23	1	165	27	5	Meningococcal disease (infants)	Increased risk	1.55(0.89-2.72)
Allen et al [60]	Gambia	198	51	2	198	51	3	Meningococcal meningitis (children)	No association	1.02(0.67-1.56)
								**Periodontitis (aggressive)**		1.04 (0.53-2.04)
Brett et al [61]	England	90	7	0	37	8	0	Aggressive periodontitis	No association	2.73 (0.96-7.76)
Emingil et al [62]	West Europe	147	7	1	86	4	0	Aggressive periodontitis	No association**^*†*^**	0.96 (0.30-3.12) [0.81(0.26-2.54)]
James et al [63]	West Europe	103	20	0	69	4	0	Aggressive periodontitis	No association	0.33 (0.12-0.97)
Noack et al [64]	Germany	71	9	0	100	11	0	Aggressive periodontitis	No association	0.86 (0.35-2.13)
Schulz et al [65]	Germany	73	7	0	52	8	0	Aggressive periodontitis	No association	1.58 (0.56-4.47)
								**Periodontitis (chronic)**		0.94 (0.75-1.18)
Garlet, et al [66]	Brazil	131	74	12	135	56	6	Chronic periodontitis	No association	0.70 (0.47-1.03)
Noack et al [67]	Germany	68	8	0	96	12	0	Chronic periodontitis	No association	1.04 (0.42-2.61)
Sahingur et al [68]	United States	59	17	1	95	19	0	Chronic periodontitis	No association	0.67 (0.33-1.37)
Schulz et al [65]	Germany	73	7	0	66	7	0	Chronic periodontitis	No association	1.10 (0.38-3.19)
Izakovicova Holla et al [69]	Czechoslovakia	195	23	0	147	24	0	Chronic periodontitis	No association	1.38 (0.76-2.53)
Berdeli et al [70]	Turkey	100	6	0	79	4	0	Chronic periodontitis	No association	0.88 (0.26-3.01)
James et al [63]	West Europe	78	16	0	77	17	1	Chronic periodontitis	No association	1.11 (0.53-2.31)
Brett et al [61]	England	90	7	0	47	6	0	Chronic periodontitis	No association	1.66 (0.55-4.97)
Laine et al [71]	Netherlands	90	8	1	90	10	0	Chronic periodontitis	No association	1.16 (0.46-2.93)
Folwaczny et al [72]	Germany	236	8	0	234	10	0	Chronic periodontitis	No association	1.24 (0.50-3.12)
								**Respiratory Syncytial Virus**		1.02 (0.72-1.44)
Lofgren, et al [73]	Finland	290	59	7	251	55	6	Respiratory Syncytial Virus	No association	1.06 (0.73-1.66)
Paulus et al [74]	Canada	97	9	0	218	17	1	Respiratory Syncytial Virus	No association	0.84(0.37-1.91)
								**Sepsis**		0.81 (0.42-1.56)
Ahmad-Nejad et al [75]	Germany	99	12	1	31	6	1	Sepsis (ICU)	No association	1.72 (0.64-4.63)
Carregaro et al [76]	Brazil	178	26	1	88	9	0	Sepsis (ICU)	No association	0.71 (0.33-1.56)
Feterowski et al [77]	Germany	135	19	0	143	10	0	Sepsis (ICU)	No association	0.51 (0.23-1.19)
								**Tuberculosis**		1.18 (0.80-1.73)
Najmi et al [78]	India	206	44	0	95	34	6	Tuberculosis	Increased association	**2.00 (1.23-3.25)**
Newport et al [79]	Gambia	235	58	5	241	62	4	Tuberculosis	No association	1.01(0.69-1.49)
Sanchez, et al [80]	Colombia	270	29	1	429	36	1	Tuberculosis	No association	0.78 (0.47-1.28)
Selvaraj et al [81]	South India	151	53	3	153	47	4	Tuberculosis	No association	0.91 (0.59-1.40)
Rosas-Taraco et al [82]	Mexico	110	4	0	94	10	0	Tuberculosis	No association	2.70 (0.87-8.39)
								**UTI**		1.41 (0.70-2.84)
Akil, et al [83]	Turkey	79	14	0	97	14	1	UTI-children	No association	0.85 (0.39-1.84)
Ertan, et al [84]	Turkey	29	1	0	28	2	0	UTI-children	No association	1.70 (0.22-13.37)
Karoly et al [85]	Hungary	218	17	0	88	15	0	UTI-children	Increased risk	2.18 (1.06-4.52)

**Table 2:** Genotypic frequencies reported for the *TLR4* 1196 C>TSNP and association with disease outcome; significant effects are in bold; outcomes that have been studied more than once have been grouped together in the table, with the overall effect described in the shaded area.

		Control Genotype				Case Genotype						
Name	Population	**C/C**		C/T	T/T	C/C	C/T	T/T	**Disease Outcome**		Conclusion Reported	OR_G_ (95% CI)
Goepfert et al [86]	United States	316		28	0	435	21	0	Bacterial Vaginosis in Pregnant		Protective	**0.55 (0.31-0.98)**
Laisk et al [33]	Estonia	287		35	1	61	9	0	*C. trachomatis*(women)		No association	1.24 (0.58-2.67)
Szebeni et al [34]	Hungary	108		10	0	37	4	0	NecEnterocolitis in LBW infants		No association	1.26 (0.39-4.00)
Lee, et al [35]	United States	395		64	4	89	18	1	Gram –ve infections in liver transplant		No association	1.23 (0.71-2.15)
Achyut et al [52]	India	188		11	1	115	9	6	H pylori		No association	2.08 (0.95-4.54)
Ajdary, et al [36]	North Iran	74		1	0	105	21	2	Leishmaniasis (Cutaneous)		Increased risk of infection	**10.14 (1.90-54.16)**
Rasouli et al [37]	Iran	137		18	0	112	9	1	Leishmaniasis (Visceral)		No association	0.67 (0.30-1.49)
Bochud et al [38]	East Africa	179		15	1	407	8	0	Leprosy		Protective	**0.23 (0.10-0.55)**
West, et al [39]	Thailand	1379		22	1	486	3	0	Meliodosis		No association**^*†*^**	0.43 (0.14-1.33) [0.41(0.13-1.25)]
Verma, et al [40]	India	140		9	1	114	25	1	Neurocysticercosis		Increased risk	**3.13 (1.46-6.73)**
Montes et al [41]	Spain	133		22	0	67	10	3	Osteomyelitis		Increased risk	1.19 (0.57-2.47)
Mrazek et al [44]	Czechoslovakia	219		33	0	88	10	0	Prosthetic joint infection		No association	0.78 (0.38-1.63)
Ahmad-Nejad et al [75]	Germany	98		13	1	31	6	1	Sepsis (ICU)		No association	1.58 (0.60-4.23)
Yuan et al [45]	Australia	365		43	1	82	3	0	S. pneumoniae		Protective	0.36 (0.12-1.09)
Liadaki, et al [46]	Greece	192		30	0	99	6	0	Tonsillar Disease (H.influenzae)		Protective	**0.42 (0.17-1.00)**
Liadaki, et al [46]	Greece	262		27	0	29	9	0	Tonsillar Disease (S.pyogenes)		Increased risk	**3.12 (1.36-7.13)**
Bhuvanendran, et al [47]	Malaysia	242		8	0	282	22	0	Typhoid Fever		Increased Risk	**2.26 (1.01-5.07)**
Hawn et al [49]	United States	277		35	4	589	69	0	UTI - Women		No association	0.83 (0.55-1.26)
									**Chagas Disease**			1.03 (0.49-2.18)
Weitzel, et al [50]	Northern Chile	42		3	0	114	11	0	Chagas Disease		No association	1.19 (0.35-4.14)
Zafra et al [51]	Colombia	282		9	0	267	8	0	Chagas disease		No association	0.95 (0.37-2.42)
									**Malaria**			1.30 (0.64-2.65)
Zakeri, et al [55]	Iran	270		50	0	271	49	0	Malaria (all ages)		No association	0.98(0.64-1.50)
Mockenhaupt et al [56]	Ghana	283		7	0	550	28	2	Malaria (pregnancy)		Overall susceptibility not studied	2.05 (0.91-4.62)
									**Periodontitis (aggressive)**			0.78(0.42-1.65)
Brett et al [61]	England	78		17	0	46	3	0	Aggressive periodontitis		No association	0.35 (0.11-1.16)
Emingil et al [62]	Turkey	148		7	0	88	2	0	Aggressive periodontitis		No association	0.57 (0.13-2.41)
Noack et al [64]	Germany	71		9	0	100	11	0	Aggressive periodontitis		No association	0.86 (0.35-2.13)
Schulz et al [65]	Germany	73		7	0	52	8	0	Aggressive periodontitis		No association	1.58 (0.56-4.47)
									**Periodontitis (chronic)**			1.12 (0.83-1.52)
Brett et al [61]	England	78		17	0	50	4	0	Chronic periodontitis		No association	0.41 (0.14-1.22)
Reddy et al [87]	South India	59		1	0	56	3	1	Chronic periodontitis		No association	2.77 (0.42-18.48)
Schulz et al [65]	Germany	73		7	0	67	7	0	Chronic periodontitis		No association	1.09 (0.38-3.14)
IzakovicaHolla et al [69]	Czechoslovakia	196		22	0	147	24	0	Chronic periodontitis		No association	1.45 (0.79-2.67)
Berdeli et al [70]	Turkey	101		5	0	80	3	0	Chronic periodontitis		No association	0.81(0.20-3.16)
James et al [63]	West Europe	74		18	0	73	20	1	Chronic periodontitis		No association	1.16 (0.58-2.32)
Noack et al [67]	Germany	68		8	0	96	12	0	Chronic periodontitis		No association	1.04 (0.42-2.61)
Laine et al [71]	Netherlands	90		8	1	90	10	0	Chronic periodontitis		No association	1.15 (0.46-2.93)
Folwaczny et al [72]	Germany	235		9	0	233	11	0	Chronic periodontitis		No association	1.22(0.51-2.93)
									**Tuberculosis**			1.07 (0.81-1.42)
Najmi et al [56]	India	206		43	1	105	26	4	Tuberculosis		No association	1.37 (0.82-2.28)
Sanchez, et al [80]	Colombia	272		26	1	429	36	1	Tuberculosis		No association	0.87 (0.52-1.46)
Selvaraj et al [81]	South India	152		46	5	150	49	4	Tuberculosis		No association	1.04 (0.68-1.61)

*†* genotypic frequencies of controls that did not satisfy Hardy Weinberg Equilibrium, [effects in brackets after correction of HWE deviations].

### TLR4 896 A>G and disease susceptibility

**Figure 1:**
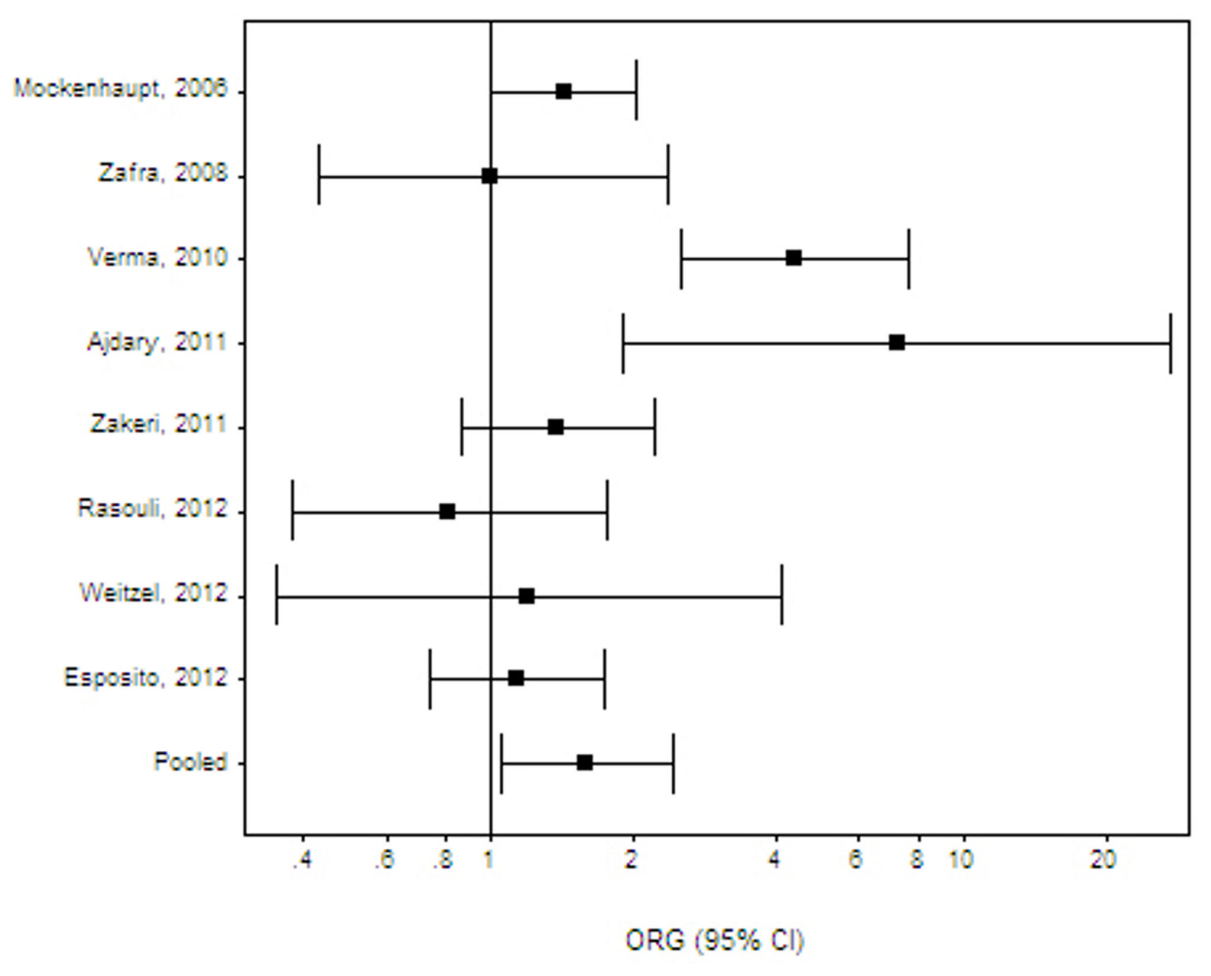
All parasitic infections: Random effects (RE) generalized odds ratio (OR_G_) estimates with the corresponding 95% confidence interval (CI) for the variant TLR4 896 A>*G*. The horizontal axis is plotted on a log scale.

**Figure 2:**
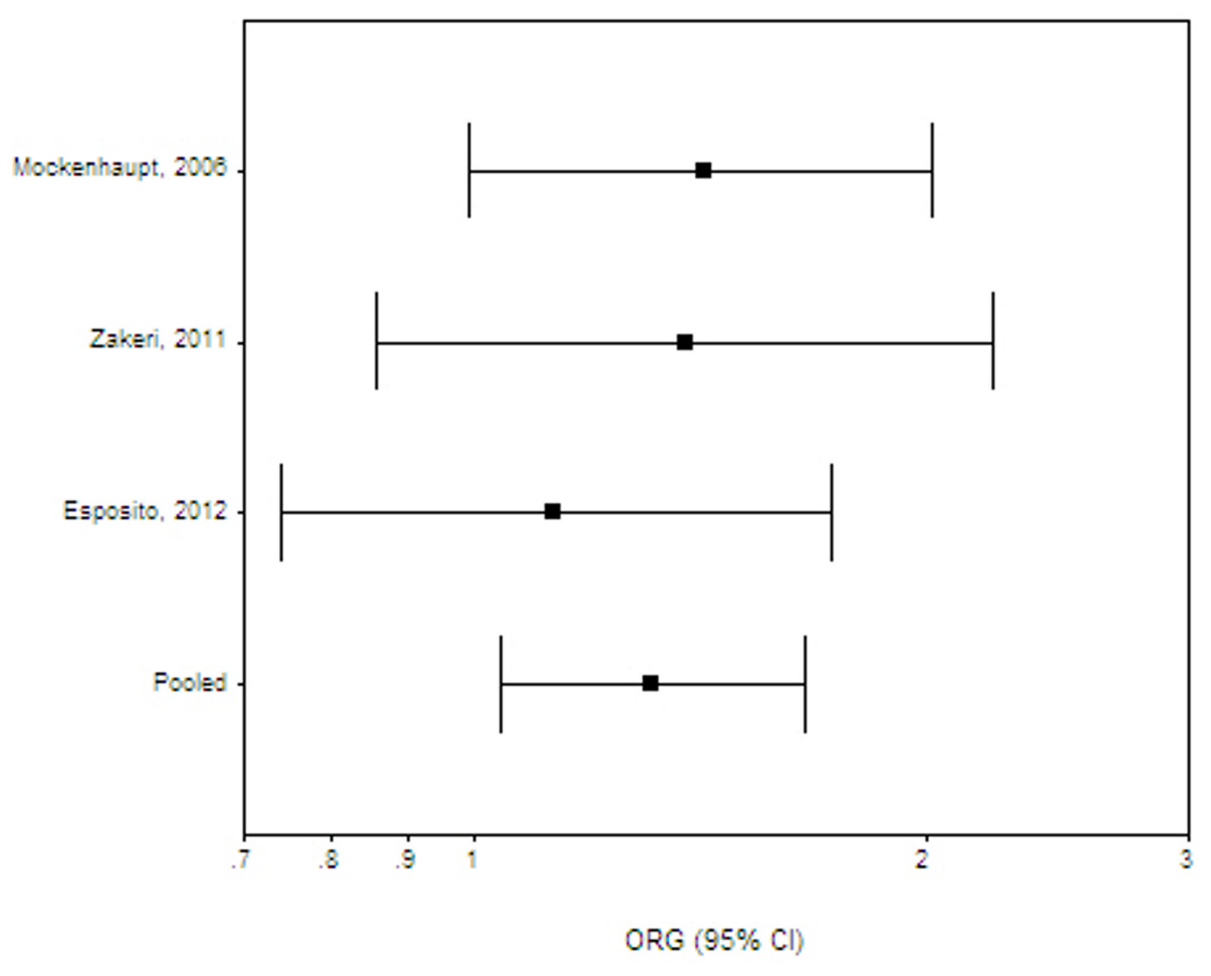
Malaria: Random effects (RE) generalized odds ratio (OR_G_) estimates with the corresponding 95% confidence interval (CI) for the variant TLR4 896 A>*G*. The horizontal axis is plotted on a log scale.

For outcomes with more than 1 available study, a meta-analysis was performed for chronic periodontitis (10 studies) [61,63,65-72], *Helicobacter pylori* infection (2 studies) [52,53], malaria (3 studies) [54-56], meningococcal disease (4 studies) [57-60], sepsis (3 studies) [75-77], respiratory syncytial virus (2 studies) [73,74], tuberculosis (5 studies) [78-82] and urinary tract infections in children (3 studies) [83-85]. Combined effects were also calculated for all Gram negative infections [30,31,33,35,39,46,47,52,53,57-60], all Gram positive infections [43,45,46] and all parasitic infections [36,37,40,50,51,54-56] (table 3). A significant risk was found for all parasitic infections combined (OR_G_ 1.59; 95% CI 1.05-2.42, effect derived from Asian, African and South American populations; Figure 1) and malaria (OR_G_ 1.31; 95% CI 1.04-1.66, a combined effect for African and Asian studies; Figure 2) . The effect on malaria was of marginal significance across African studies [54,56] (OR_G_ 1.29; 95% CI 0.99-1.69). All other effects were insignificant, namely all Gram negative infections (OR_G_ 1.10; 95% CI 0.90-1.38), all Gram positive infections (OR_G_ 1.28; 95% CI 0.43-3.81), Chagas disease (OR_G_ 1.06; 95% CI 0.53-2.14) , *H. pylori* (OR_G_ 0.91; 95% CI 0.61-1.36), meningococcal disease (OR_G_ 1.10; 95% CI 0.90-1.34), aggressive or chronic periodontitis (OR_G_ 1.04; 95% CI 0.53-2.04 and OR_G_ 0.94; 95% CI 0.75-1.18, respectively), respiratory syncytial virus (OR_G_1.02; 95% CI 0.72-1.44), sepsis (OR_G_ 0.81; 95% CI 0.41-1.56) and tuberculosis (OR_G_ 1.18; 95% CI 0.80-1.73). The meta-analysis results are summarized in Table 3. Statistical heterogeneity varied from absent to moderate. The Harbord’s test indicated that there is no differential magnitude of effect in large versus small studies for all outcomes (p≥0.05). Across populations of European ancestry, the risk of meningococcal disease [57-59] (OR_G_ 1.12; 95% CI 0.85-1.49) and chronic periodontitis (excluding the two non-European studies [66,68]; OR_G_ 1.06; 95% CI 0.53-2.14) remained insignificant. The effects on meningococcal disease and aggressive periodontitis did not alter after removing from analysis the two studies not in HWE equilibrium (data not shown) [58,62]. Effects on tuberculosis remained insignificant across Indian [78,81] (OR_G_ 1.34; 95% CI 0.62-2.90) or S. American [80,82] populations (OR_G_ 1.30; 95% CI 0.39-4.33). For outcomes with a single available study, a significant risk was present for brucellosis (OR_G_ 2.66; 95% CI 1.66-4.27) [30], cutaneous leishmaniasis (OR_G_ 7.22; 95% CI 1.91-27.29) [36], neurocysticercosis (OR_G_ 4.39; 95% CI 2.53-7.61) [40], and typhoid fever (OR_G_ 2.51; 95% CI 1.18-5.34) [47]. All the significant single-study effects are summarized in Table 4.

**Table 3:** Summary of disease associations derived from meta-analysis of case-control studies.

**Disease Outcome**	**Studies**	**Polymorphism**	**Effect (OR_G_ ; 95% CI)**	**P_Q_**	**I^2^**	**P_H_**
All Gram - infections	13	*TLR4 896 A>G*	1.10 (0.90-1.38)	0.01	52%	0.32
	6	*TLR4 1196 C>T*	1.11 (0.66-1.87)	0.02	61%	0.59
*Helicobacter pylori*	2	*TLR4 896 A>G*	0.91 (0.61-1.36)	0.79	-	-
Meningococcal Disease	4	*TLR4 896 A>G*	1.10 (0.90-1.34)	0.43	0	0.93
All Gram + infections	3	*TLR4 896 A>G*	1.28 (0.43-3.81)	0.01	77%	-
	2	*TLR4 1196 C>T*	1.09(0.13-9.09)	0.002	-	-
All parasitic infections	8	*TLR4 896 A>G*	1.59 (1.05-2.42)	<0.001	72%	0.72
	7	*TLR4 1196 C>T*	1.50 (0.88-2.56)	0.01	64%	0.5
Chagas Disease	2	*TLR4 896 A>G*	1.06 (0.53-2.14)	0.82	-	-
	2	*TLR4 1196 C>T*	1.03 (0.49-2.18)	0.76	-	-
Malaria	3	*TLR4 896 A>G*	1.31 (1.04-1.66)	0.71	0	-
	2	*TLR4 1196 C>T*	1.30 (0.64-2.65)	0.11	-	-
Periodontitis(Aggressive)	5	*TLR4 896 A>G*	1.04 (0.53-2.04)	0.07	52%	0.16
	4	*TLR4 1196 C>T*	0.78 (0.42-1.65)	0.29	20%	0.92
Periodontitis (Chronic)	10	*TLR4 896 A>G*	0.94 (0.75-1.18)	0.68	0	0.74
	9	*TLR4 1196 C>T*	1.12 (0.83-1.52)	0.74	0	0.93
RSV	2	*TLR4 896 A>G*	1.02 (0.72-1.44)	0.61	-	-
Sepsis	3	*TLR4 896 A>G*	0.81 (0.41-1.56)	0.16	45%	-
Tuberculosis	5	*TLR4 896 A>G*	1.18(0.80-1.73)	0.03	63%	0.43
	3	*TLR4 1196 C>T*	1.07 (0.81-1.42)	0.47	0	-
UTI (Children)	3	*TLR4 896 A>G*	1.41 (0.70-2.84)	0.21	35%	-

P_Q=_ p value for Q homogeneity test; P_H_= p value for Harbord’s small study effects test, -=not applicable

**Table 4:** Summary of significant associations with disease outcomes, derived from single case-control studies.

**Study**	**Population**	**Disease Outcome**	**Polymorphism**	**OR_G_ (95% CI**)
Goepfert [86]	USA	Bacterial vaginosis (pregnancy)	*TLR4 1196 C>T*	0.55 (0.31-0.98)
Rezazadeh[30]	Iran	Brucellosis	*TLR4 896 A>G*	2.66 (1.66-4.27)
Ajdary [36]	Iran	Cutaneous leishmaniasis	*TLR4 896 A>G*	7.22 (1.91-27.29)
			*TLR4 1196 C>T*	10.14 (1.90-54.16)
Bochud [38]	East Africa	Leprosy	*TLR4 896 A>G*	0.36 (0.22-0.60)
			*TLR4 1196 C>T*	0.23(0.10-0.55)
Verma [40]	India	Neurocysticercosis	*TLR4 896 A>G*	4.39 (2.53-7.61)
			*TLR4 1196 C>T*	3.13 (1.46-6.73)
Liadaki [46]	Greece	*H.influenzae* (tonsillitis)	*TLR4 1196 C>T*	0.42 (0.17-1.00)
Liadaki [46]	Greece	*S.pyogenes* (tonsillitis)	*TLR4 896 A>G*	2.93 (1.24-6.93)
			*TLR4 1196 C>T*	3.12 (1.36-7.13)
Bhuvanedran [47]	Malaysia	Typhoid fever	*TLR4 896 A>G*	2.51 (1.18-5.34)
			*TLR4 1196 C>T*	2.26 (1.01-5.07)
Yin [48]	China	UTI (Adults)	*TLR4 896 A>G*	1.98 (1.04-3.98)

Of note, all these effects were derived from Asian studies. Increased risk for tonsillar infection due to *Streptococcus pyogenes* (OR_G_ 2.93; 95% CI 1.24-6.93) [46] was noted in the Greek pediatric population, as was an increased risk for urinary tract infections in adults (OR_G_ 1.98; 95% CI 1.04-3.98) in a Chinese population [48]. Interestingly, not all outcomes were negative and the *TLR4* 896 A>*G* polymorphism was associated with significant protection against leprosy (OR_G_ 0.36; 95% CI 0.22-0.60) in East Africa [38].

The use of the OR_G_ metric resulted in more conservative estimates of associations, as two reportedly significant associations (1 reporting increased risk for Gram-negative osteomyelitis [41] and 1 reporting a protective effect for *Streptococcus pneumoniae* in children [45]) were downgraded to non-significant. Six control populations deviated for HWE equilibrium [30,35,39,53,58,62], and associations of TLR4 variants with disease were readdressed after correcting genotypes with their expected frequencies. These effects did not change (they appear in brackets in Tables 1,2). Specifically, the association of *TLR4* 896 A>*G* and brucellosis [30] remained significant after HWE correction (OR_G_ 2.69; 95% CI 1.67-4.33).

### TLR4 1196 C>T and disease susceptibility

A meta-analysis of GAS was performed for malaria (2 studies) [55,56], aggressive periodontitis (4 studies) [61,62,64,65], chronic periodontitis (9 studies) [61,63,65,67,69-72,87], and tuberculosis (3 studies) [78,80,81] and revealed no significant effects. Statistical heterogeneity varied from absent to moderate. Specifically, the combined effects were OR_G_ 1.30 (95% CI 0.64-2.65) for malaria, OR_G_ 0.78 (95% CI 0.42-1.65) for aggressive and OR_G_ 1.12 (0.83-1.52) for chronic periodontitis, and OR_G_ 1.07 (95% CI 0.81-1.42) for tuberculosis. Effects were also insignificant for all Gram negative infections combined [OR_G_ 1.11 (95% CI 0.66-1.87)][33,35,39,46,47,52], all Gram positive infections combined [OR_G_ 1.09 (95% CI 0.13-9.09)] [45,46] and all parasitic infections combined [OR_G_ 1.50 (95% CI 0.88-2.56)] [36,37,40,50,51,55,56]. The meta-analysis results are summarized in Table 3. The Harbord’s test indicated that there is no differential magnitude of effect in large versus small studies for all outcomes (p≥0.05).

For outcomes with a single available study, a significant risk was present for cutaneous leishmaniasis in Iran (OR_G_ 10.14; 95% CI 1.90-54.16) [36], neurocysticercosis in India (OR_G_ 3.10; 95% CI 1.45-6.67) [40], *S. pyogenes* tonsillar disease in Greece (OR_G_ 3.12; 95% CI 1.36-7.13) [46] and typhoid fever in Malaysia (OR_G_ 2.26; 95% CI 1.01-5.07) [47]. A significant protection was conferred for bacterial vaginosis in pregnancy (OR_G_ 0.55;95% CI 0.31-0.98) in the United States (notably, African Americans comprised 78% of the cases) [86], leprosy in East Africa (OR_G_ 0.23; 95% CI 0.10-0.55) [38], and *Haemophilus influenzae* tonsillar disease in a Greek pediatric population (OR_G_ 0.42; 95% CI 0.17-1.00) [46]. The significant results are summarized in Table 4. Only 1 control population deviated from HWE equilibrium that assessed the risk of meliodosis [39], a risk that did not change after correction with the expected genotype frequencies (Table 2). Two reportedly significant associations for Gram-negative osteomyelitis (increased risk) and *S. pneumoniae* (protection) were not confirmed in this analysis with the use of the OR_G_ metric. 

The significant effects were unidirectional and similar in magnitude when both *TLR4* 896 A>*G* and 1196 C>*T* were examined (Table 4), that is if *TLR4* 896 A>*G* was protective then 1196 C>*T* was also protective. When *TLR4* 896 A>*G* increased risk, then 1196 C>*T* increased risk. Specifically, the point estimates for 896 A>*G* and 1196 C>*T* variants were (respectively): 7.22 and 10.14 for cutaneous leishmaniasis, 4.39 and 3.13 for neurocysticercosis, 2.93 and 3.12 for *S. pyogenes* tonsillar disease, 2.51 and 2.26 for typhoid fever, 0.36 and 0.23 for leprosy. An exception to the rule was *H. influenzae* tonsillar disease, where the protective effect of *TLR4* 896 A>*G* did not reach statistical significance (OR_G_ 0.47; 95% CI 0.19-1.14), while 1196 C>*T* showed significant association (OR_G_ 0.42; 95% CI 0.17-1.00).

## Discussion

We performed a systematic literature review to address the potential association of 2 common TLR4 single nucleotide polymorphisms (*TLR4 896 A>G, TLR4 1196 C>T*) with infectious diseases. An increased risk was documented for all parasitic infections combined, malaria [54-56], brucellosis [30], cutaneous leishmaniasis [36], typhoid fever [47], neurocysticercosis [40] and adult urinary tract infections [48]. Interestingly, all these effects were reported in populations of Asian descent, with the exception of parasitic infections and malaria where the effect was a combined effect from Asian, African and South American populations. This finding is more striking when we consider that European populations comprised the majority of GAS data (28 out of 59 studies, 48%) and a significant risk was found only for TLR4 polymorphisms and *S. pyogenes* tonsillitis among Greek children [46]. Another notable finding is that, for some infections, these single nucleotide polymorphisms were associated with lower infection rates. Overall, these effects sum to a total of 11 significant SNPs-disease associations that represent almost one third (30%) of all outcomes addressed in the eligible studies and there was consistency of effects (risk or protection) between 896 A>*G* and 1196 C>*T* variants when both associations were studied. 

In this study we utilized the generalized odds ratio (OR_G_) metric to quantify the magnitude of associations. This metric provides a straightforward interpretation of the relative risk effect, based solely on genotype distribution [20]. The generalized odds ratio overcomes this problem by directly quantifying the magnitude of association of a gene with disease [20]. Implementing the OR_G_ obviates the need for selecting, estimating and interpreting individual genotype contrasts (dominant, recessive and co-dominant) and their effect. OR_G_ can also be used in meta-analysis of GAS to summarize effects and produce robust results, avoiding the shortcomings of multiple model testing, namely the lack of biologic justification and non-independency of effects [20,88,89]. For example, for *TLR4* 896 A>*G* association with malaria, the combined OR_G_ showed that the probability of having malaria might be 31% higher for subjects having higher mutational load relative to those with lower mutational load (subjects who are homozygous for *G* allele have the highest mutational load, those homozygous for A allele have the lowest, and heterozygous have an intermediate level). The application of the OR_G_ metric also resulted in a more conservative estimate of associations, given that associations for infections such as osteomyelitis (39) and *S. pneumonia* (43) were downgraded to insignificant. The associations derived from tuberculosis data were insignificant similar to those reported [90].

In our analysis, TLR4 polymorphisms were associated with susceptibility to a diverse spectrum of infections including Gram-negative, Gram-positive bacteria as well as parasitic infections, such as cutaneous leishmaniasis and neurocysticercosis. This wide spectrum of associations correlates with the spectrum of recognition molecules by TLR4. Indeed, TLR4 is involved in induction of cell-mediated immunity to *Brucella abortus* in mice [7] and TLR4 signaling also upregulates macrophage anti-leishmanial activity [91]. Similarly, binding of the *Salmonella typhi* porin OmpS1 to TLR4 leads to overexpression of MHCII and CD40 molecules and activation of dendritic cells [92]. TLR4 can recognize LPS of Gram-negative bacteria [3,4], glycans of the helminth *Taenia solium* [93] as well as the fusion protein of respiratory syncytial virus[9].

Interestingly, our analysis also confirmed that these polymorphisms are also protective for certain types of infection, such as leprosy. It is not clear why such polymorphisms confer increased susceptibility to some infection, but protect from others. It could be speculated that in some infections the immune response leads to an inflammatory response that is protective, whereas in others such response may be essential in the pathogenesis of the infectious process. An example is *Mycobacterium leprae* where the TLR4-mediated immune response to the pathogen may modulate inflammatory processes that influence disease manifestations but are not attributable to direct stimulation by M. leprae. Indeed, Bochud et al [38] found that the stimulation of monocytes with M. leprae inhibited their subsequent response to TLR4 stimulation with LPS.

Among Indo-European populations, 6-14% of the individuals are double heterozygous for both polymorphisms [94]. It is suggested that the double heterozygous *TLR4* 896 A>*G*/TLR4 1196 C>*T* haplotype does not functionally differ from wild type TLR4. Therefore, co-segregation may result in a functionally neutral phenotype and, as seen in European populations, lead to the lack of significant associations. Conversely, *TLR4* 896 A>*G* was frequently found (10-18%) among African populations, with only 2% having *TLR4* 1196 C>*T* co-segregation. Two studies (on typhoid fever and leprosy) indicated weak linkage disequilibrium in Malaysian [47] and East African populations [38]. These differences between Europeans (co-segregation) compared to Asian and African population (lack of co-segregation) may explain why the majority of significant associations were noted for endemic diseases of Asia and Africa. 

Our analysis on the impact of these polymorphisms in periodontitis illustrates the different impact of polymorphisms based on the population. More specifically, despite the bulk of studies on aggressive and chronic periodontitis, TLR4 variants did not show any significant association, even though TLR4 has been shown to be overexpressed in gingival epithelial cells and gingival fibroblasts [95-97] in association with periodontal inflammation involving pathogens related to periodontitis, such as *Porphyromonas gingivalis*, *Fusobacterium nucleatum* and *Aggregatibacter actinomycetemcomitans* [98-101]. One possible explanation is that this finding was because all relevant studies were almost exclusively confined to European ancestry populations and the lack of susceptibility may be related to the strong linkage disequilibrium, that is the non-random association between 896 A>*G and* 1196 C>*T* in Europeans [94]. 

Importantly, our analysis highlights the need to evaluate the impact of these polymorphisms in different populations and various clinical conditions. Moreover, the absence of significant associations in meta-analysis data for periodontitis, tuberculosis, meningococcal disease and sepsis, signifies that the functional alterations related to polymorphic TLR4 variants may not be critical to produce the clinical phenotype. Lack of reproducibility stands as a barrier for conclusive evidence, and design, sample size and environmental and genetic heterogeneity between populations may affect results. Finally, the presence of a significant effect may rely on the magnitude of functional expression of TLR4. Protection or risk may be moderated by the level of TLR4 functional expression, which is modulated by TLR4 polymorphism and MD-2 presence [14,15]. Therefore, it is essential to explore whether MD-2 is important in the response to some infections, but not others, or that levels of TLR4 vary in one infection compared to another.

The heterogeneity of the populations studied along with multiple endpoints should also be considered as potential study limitation that may influence statistical power. Moreover, different populations mount diverse immunologic responses and the clinical relevance of polymorphisms is not always straightforward. The lack of association for a disease phenotype highlights that gene-to-gene interactions and gene-environment interactions may be influential parameters of disease association. Case-control design of individual GAS precludes adjusted analysis for gene-gene-environment interactions and may have reduced the efficiency of genetic risk estimates, though it is unlikely to inflate false-positive results [89].

Despite these limitations, genetic markers of immune response such as TLR4 variants, are valuable not only to classify high-risk patients based on disease susceptibility but also to predict disease severity and other sequelae. The associations of TLR4 896A>G with hearing loss in survivors of bacterial meningitis [102] and the increased risk of tympanostomy among toddlers with history of bronchiolitis [103] are indicative examples.

 In conclusion, our analysis highlights the complex effect of TLR variants in susceptibility to infectious disease. Some of the effects, such as in malaria, are validated in a variety of studies, whereas single case-control studies should be cautiously interpreted until more information on the specific outcomes is added. Taken in their totality, our results indicate that depending on the infection and the population studied, the same polymorphism may be associated with risk, protection or have no effect. In this context, our analysis provides the rationale for understanding the protective or adverse effect of TLR4 polymorphisms and may provide a basis to explain the maintenance of these polymorphisms.

## Supporting Information

Checklist S1 **PRISMA checklist.**
(DOC)

Table S1 **Flow diagram of meta-analysis.**
(DOCX)
