# Supplementary material for: The Role of TLR4 896 A>G and 1196 C>T in Susceptibility to Infections: A Review and Meta-Analysis of Genetic Association Studies
Source: PLoS One. 2013 Nov 25;8(11):e81047. doi: 10.1371/journal.pone.0081047 (PMC3840016; doi:10.1371/journal.pone.0081047)
Supplement: Table S1 — Flow diagram of meta-analysis. (DOCX) [file pone.0081047.s002.docx]

**Flow diagram of meta-analysis**

Potentially relevant studies identified and screened for retrieval
PubMed (n = 962), EMBASE (n = 1615)

Studies included in the analysis
(n= 59)

Studies added after manual search of reference lists of included studies (n=0)

Studies to be included in the meta-analysis (n = 117)

Studies excluded (n= 58) after full-text evaluation
18 studies did not publish genotypic frequencies
13 studies had no healthy controls
5 studies were in vitro functional studies
8 studies did not study the desired polymorphisms
6 studies were reviews/meta- analysis
8 studies had non extractable data for other reasons

Studies included in the analysis
(n= 59)

Duplicate studies excluded (n=380)

Non-duplicate studies for evaluation (n=2197)

Studies excluded after title and abstract reading (n=2080)
